# Effects of *Paeonia lactiflora* Extract on Estrogen Receptor *β*, TPH2, and SERT in Rats with PMS Anxiety

**DOI:** 10.1155/2020/4690504

**Published:** 2020-03-07

**Authors:** Jieqiong Wang, Chunhong Song, Dongmei Gao, Sheng Wei, Wenjun Sun, Yinghui Guo, Shiguang Sun, Xi Tian, Huihao Li, Mingqi Qiao

**Affiliations:** ^1^School of Pharmacy, Shandong University of Traditional Chinese Medicine, Jinan, China 250355; ^2^Experimental Center, Shandong University of Traditional Chinese Medicine, Jinan, China 250355; ^3^School of Chinese Medicine, Shandong University of Traditional Chinese Medicine, Jinan, China 250355; ^4^Drug Room, Second Affiliated Hospital, Shandong University of Traditional Chinese Medicine, Jinan, China 250355

## Abstract

This study is to investigate the effect of *Paeonia lactiflora* extract on PMS anxiety and on expression of estrogen receptor *β* (ER*β*), tryptophan hydroxylase-2 (TPH2), and serotonin transporter (SERT) in the premenstrual syndrome (PMS) anxiety model rats. The vaginal smear and open field test were used to screen rats in nonreception phase of estrus cycle with similar macroscopic behaviors and regular estrus cycle. PMS anxiety model rats were prepared by electrical stimulation. RT-PCR and immunofluorescence were used to measure the expression of ER*β*, TPH2, and SERT. Compared with normal rats, the total distance in the open field test of the model rats was significantly increased (*P* < 0.05). The model rats showed nervous alertness, irritability, and sensitivity to external stimuli. After treatment with the *Paeonia lactiflora* extract, the total distance of rats was significantly reduced (*P* < 0.05). In reception stage, there was no significant difference in the mRNA and protein expression of ER*β*, TPH2, and SERT. In nonreception stage, the expression of ER*β* and TPH2 in the model group was significantly decreased (*P* < 0.05) as compared with the control group, but not SERT. Abnormal changes of the above indicators were reversed after the administration of the *Paeonia lactiflora* extract. In conclusion, *Paeonia lactiflora* extract can increase the expression of ER*β* and TPH2 and decrease SERT in PMS model rats, which may be one of the mechanisms underlying the effect of *Paeonia lactiflora* extract on PMS.

## 1. Introduction

Premenstrual syndrome (PMS) refers to periodic symptoms of somatic, mental, and behavioral symptoms during the luteal phase of the menstrual period in women of childbearing age. It is characterized by the natural disappearance of symptoms within a few days of menstrual cramps and belongs to the category of emotional disorders [1]. The main symptoms are premenstrual upset, irritability, and anxiety [1].

The pathogenic factors of PMS are complex and diverse [2]. Study shows that the decline of estrogen may be one of the central mechanisms in the pathogenesis of PMS and is especially associated with the premenstrual anxiety and irritability symptoms [2]. Borrow and Cameron also believed that the occurrence of negative emotions in PMS was closely related to changes in estrogen, which can regulate emotional activities by regulating serotonin (5-HT) neurons in the central nervous system [3]. Robichaud and Debonnel [4] have shown that there were estrogen receptors (ERs) in the 5-HT neurons in nonhuman primates. Clinical study suggests that the regulation of emotions by ERs is mainly through the 5-HTergic system [5]. Imwalle et al. [6] reported that when the ER*β* gene was knocked out, the content of 5-HT in the hippocampus and the nucleus raphe magnus was significantly reduced as compared with wild-type rat, indicating that estrogen may increase 5-HT levels in the brain. At the same time, they also found that the ER*β*-deficient rat exhibited increased anxiety behavior in the ascending cross-maze experiment. Thus, estrogens can regulate the synthesis, conduction, and reabsorption of 5-HT through the ER, thereby exerting the effect of emotion regulation.

Tryptophan hydroxylase (TPH) and serotonin transporter (SERT) are involved in the synthesis and metabolism of the 5-HTergic system, respectively. TPH is the only rate-limiting enzyme in the 5-HT synthesis. It is reported that the decrease of 5-HT content in the hippocampus of rats with ovariectomy was due to the decreased expression of TPH2 in the nucleus raphe [5]. The main function of SERT in the 5-HTergic system is to recapture the 5-HT in the synaptic cleft into cells for degradation or recycling. SERT expression changes directly affect the neural activity involved in 5-HTergic neurons.

Charoenphandhu et al. [7] also demonstrated that 17*β*-estradiol could treat the anxiety of ovariectomized rats through upregulation of *SERT* mRNA. Donner and Handa [8] also confirmed that the expression of *TPH2* mRNA in the tail and middle of rat dorsal raphe nucleus (DRN) was increased after injection of ER*β* agonist once a day for 8 consecutive days in ovariectomized rats. On the 6^th^ and 7^th^ days of treatment, both open field and elevated maze experiments demonstrated that ER*β* had anxiolytic-like properties. On the contrary, Bethea et al. [9] concluded that the expression of *SERT* mRNA in ovariectomized rhesus macaques after 17*β*-estradiol treatment was significantly decreased as compared with the control group. Pecins-Thompson et al. [10] used estrogen therapy on Ovx rhesus monkeys for 28 days and revealed that estrogen treatment reduced the level of *SERT* mRNA in DRN by approximately 32%. Meanwhile, *SERT* mRNA-positive cells were also significantly reduced. Zhou et al. [11] reported that after chronic estrogen administration to ovariectomized rats, *SERT* mRNA was significantly reduced in the middle part of rat DRN. These findings imply that PMS anxiety may be closely related to estrogen-mediated expression of TPH2 and SERT in the 5-HTergic system of DRN. However, the results are controversial.

The total glucosides of paeony are the main extracts of *Paeonia lactiflora*. Paeoniflorin, the main active ingredient of glucosides of paeony, has sedative effect and can improve learning and memory, regulate the hypothalamic-pituitary-adrena axis, and increase the stress ability and anxiolytic ability of rats [12, 13]. Paeonimetabolins I, a major metabolite of Paeoniflorin, has anticonvulsant and anxiolytic-like effects [14]. Takeuchi et al. [15] showed that paeoniflorin promoted E_2_ synthesis by direct action on estrus ovary in rats. It is also reported that paeoniflorin could reverse the reduction of 5-HT and 5-hydroxyindoleacetic acid (5-HIAA) levels in the prefrontal cortex and hippocampus, respectively, and exert antidepressant effects [16]. Qiu et al. [17] reported that the upregulation of 5-HT and noradrenergic systems was an important mechanism underlying the antidepression-like effects of paeoniflorin in rats with chronic stress depression. These indicate that paeoniflorin may have antidepression and antianxiety effects via regulating E_2_ and 5-HTergic systems.

In this study, the effects of *Paeonia lactiflora* on PMS anxiety and on the expressions of ER*β*, TPH-2, and SERT in DRN of rats with PMS anxiety were investigated. The underlying mechanisms were also analyzed and discussed.

## 2. Materials and Methods

### 2.1. Animals

Healthy female nonpregnancy Wistar rats (SPF grade) weighing 150-180 g (*n* = 120) were provided by Beijing Vital River Laboratory Animal Technology Co., Ltd (license number SCXK (Beijing) 2007-0001). All experimental procedures involving animals were conducted according to the ethical guidelines of Shandong University of Traditional Chinese Medicine. All efforts were made to minimize animal suffering.

### 2.2. Animal Grouping and PMS Model Establishment

**Figure 1:**
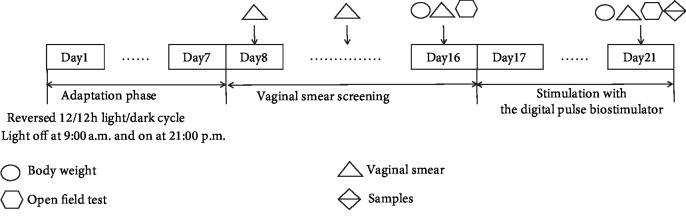
Experimental flowchart of PMS model establishment. Time points of the body weight measurements (circles), the open field test (hexagon), the vaginal smear test (triangles), and the sample collection (double triangles) are shown. Vaginal smear method was used to select the rats with regular estrous cycles (a cycle for 4 to 5 days). Then, these rats were weighed and subjected to open field test. After that, they were grouped. Each group contained both rats in the reception phase and nonreception phase. In the following estrus cycle, PMS modeling was carried out and corresponding drugs were given. After modeling, samples were collected.

The experimental design was shown in Figure 1. All animals were housed under a day-and-night reversed environment (12/12 h light/dark cycle; lights off at 9 : 00 a.m. and light on at 21 : 00 p.m.). Vaginal smear and open field test were used to screen out rats with regular estrus behavior [18]. Finally, 120 rats with regular estrous cycles were randomly divided into 5 groups: control group, PMS model group, *Paeonia lactiflora* extract group, fluoxetine group, and ER*β* agonist group, with 24 rats in each group. Except for rats in the control group, liver-qi invasion syndrome of PMS model was established in the other four groups. Briefly, rats were stimulated with a ST-A digital pulse biostimulator (jointly developed by the Jinan Air Force Logistics Assembly Plant and Shandong University of Traditional Chinese Medicine) for 5 days. The electrical simulation parameters included 2700 to 3300 v of voltage, 0.3 s of pulse width, and 5 min of pulse interval during the day and 10 min at night. The electrical stimulation was accompanied by noise stimulation. At the same time, *Paeonia lactiflora* extract and fluoxetine groups were administrated by gavage with 40 mg/kg of *Paeonia* lactiflora extract (Herb-Key, Xi'an, China) and 2.67 mg/kg of fluoxetine (Eli Lilly and Company, Suzhou, China) daily for 5 days, respectively. ER*β* agonist group were injected subcutaneously with 2 mg/ml ER*β* agonist DPN (Abcam, UK) at a dose of 2 mg/kg daily for 5 days. Control and PMS model groups received equal volume of sterile water daily for 5 days.

After 5 days, brain samples were collected from each group, including 6 rats in the reception phase of estrus cycle and 6 rats in nonreception phase of estrus cycle. Briefly, the rats were anesthetized with 1% sodium pentobarbital (0.8 ml/200 g) and subjected to cardiac perfusion, decapitation, and brain sampling. The brain tissues and the dorsal nucleus of the brain tissues were isolated and stored at -70°C.

### 2.3. Vaginal Smear

**Figure 2:**
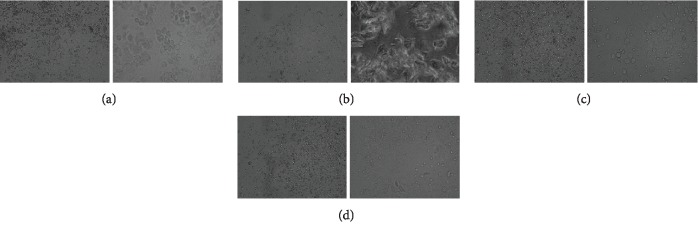
Photomicrograph of cells in each phase of rat estrus cycle. (a) Preestrus. Vaginal smears included mostly nucleated epithelial cells, occasionally with a small amount of keratinization, and no leukocyte. (b) Midestrus. Vaginal smears included mostly nucleated keratinocytes, a small amount of epithelium, and no white blood cells. (c) Late estrus. Vaginal smears included keratinocytes, nucleated epithelial cells, and white blood cells. (d) Diestrus. Vaginal smears included a large number of white blood cells and a small amount of epithelial cells and mucous cells. Left panel, 10×; right panel, 40×.

The rat was held firmly. The saline (20 *μ*l) was slowly injected into the rat's vagina. After a few seconds, the liquid was slowly drawn from the vagina, smeared onto the slide, and observed under a microscope (CX21BIM-SET5, OLYMPUS, Tokyo, Japan). In this experiment, the rats were examined for 10 consecutive days. Finally, the nonreception phase of estrus cycle rats with white blood cells in vaginal smear were in the nonreception phase of estrus cycle and were selected for modeling experiment. The cell morphology of each phase of estrus cycle was shown in Figure 2.

### 2.4. Open Field Test

The rats were placed in a field with a length and width of 50 cm x 50 cm. The side wall of the field was 40 cm high and the color of the bottom surface was black. The field was divided into the peripheral area and the central area, which was about 1/3 of the entire field. The Xmaze animal behavior analysis system was used to track the total distance of travel in the open field within 3 minutes. To avoid odor interference between different rats, the field was thoroughly cleaned at the end of the test for each rat with 75% ethanol.

### 2.5. Reverse Transcription PCR

Total RNAs were extracted from the brain tissues with Trizol Total RNA Extraction Kit (Sangon, Shanghai, China). Then, RNA was reverse transcribed into cDNA. The mRNA expression levels of *TPH2, ERβ*, and *SERT* were detected. *β*-Actin was used as an internal reference. Primers used in this study were synthesized by Sangon (Shanghai, China) and primer sequences were given in Table 1. The PCR procedures for *TPH2* were 94°C for 2 min 30 s followed by 33 cycles of 94°C for 30 s, 59.3°C for 40 s, 72°C for 1 min, and a final extension at 72°C for 7 min. The PCR procedures for *ERβ* were 94°C for 2 min and 30 s followed by 34 cycles of 94°C for 30 s, 61.6°C for 40 s, 72°C for 1 min, and a final extension at 72°C for 7 min. The PCR procedures for *SERT* were 94°C for 2 min and 30 s followed by 30 cycles of 94°C for 30 s, 61°C for 40 s, 72°C for 1 min, and a final extension at 72°C for 7 min. The PCR procedures for *β*-actin were 94°C for 2 min and 30 s followed by 28 cycles of 94°C for 30 s, 59°C for 40 s, 72°C for 1 min, and a final extension at 72°C for 7 min. The amplified products were electrophoresed on 1.2% agarose gel, and the image was analyzed by the gel image analysis system FR-980 (Furi, Shanghai, China). The gray ratio of each gene to *β*-actin was used as the relative expression of the gene.

### 2.6. Immunofluorescence

Brain tissues were fixed in paraformaldehyde, embedded, and cut into sections (5 *μ*m thickness). Then tissue sections were dewaxed in xylene and rehydrated in graded alcohols. After that, sections were incubated with 0.3% hydrogen peroxide to inactivate endogenous peroxidase activity. Antigen retrieval was achieved by incubating with 0.1 M sodium citrate (pH 6.0). After blocking with goat serum (Cat# ZLI-9022; Zhong Shan-Golden Bridge Biological Technology CO., Ltd., Beijing, China) for 30 min, sections were incubated with primary antibodies against TPH (1 : 300; ab46200; Abcam), ER*β* (1 : 200; ab3577; Abcam), and SERT (1 : 1000; ab44520; Abcam) at 4°C overnight. After washing with PBS, secondary antibodies of FITC tagged goat anti-rabbit IgG (1 : 100; ZF-0311, ZSGB-bio, Beijing) were added and incubated for 30 minutes in the dark. After rinsing with PBS for 3 times, the sections were analyzed on a laser scanning confocal microscope (LSM510, Zeiss, Jena, Germany). Three different visual fields were randomly selected for each target brain area of each slice for photographing. The fluorescence intensity was quantified using Image-Pro Plus 6.0 (Media Cybenetics, Silver Spring, USA).

### 2.7. Statistical Analysis

Statistical analyses were performed using GraphPad Prism 5 (GraphPad Software, Inc., Cary, NC). The results were expressed as mean ± SD. The one-way ANOVA was used to compare the differences among groups. Bonferroni post hoc tests were performed following ANOVA where appropriate. A *P* value less than 0.05 was considered statistically significant.

## 3. Results

### 3.1. Effect of *Paeonia lactiflora* Extract on the Field Behavior

**Figure 3:**
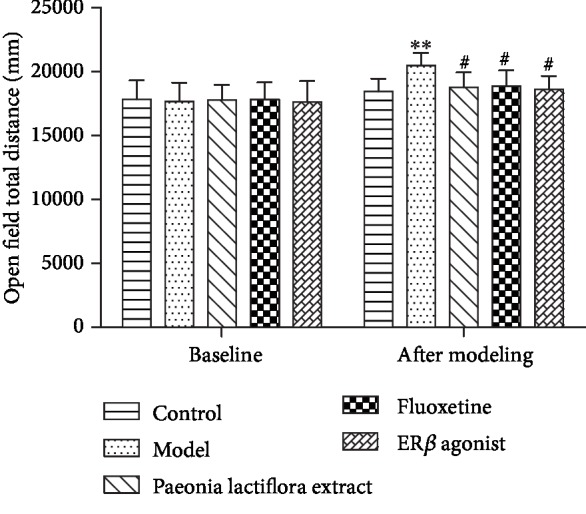
Column chart of the total distance of rats in each group before and after modeling. ^∗∗^*P* < 0.01 as compared with the normal group; ^#^*P* < 0.05 as compared with the model group.

In this study, open field test was used to determine whether the preparation of model rat with liver-qi reverse of PMS was successful. As shown in Figure 3, the total distance of rats in the model group increased significantly as compared with control group (*P* < 0.01), indicating a higher level of autonomic movement and exploration activities in the model group. Compared with the model group, the total distance of rats decreased significantly (*P* < 0.05) after treatment with the *Paeonia lactiflora* extract. These results indicate that the rats in the PMS model group showed obvious anxiety state, and the model was successfully prepared. In addition, the *Paeonia lactiflora* extract administration could significantly improve anxiety.

### 3.2. Effect of *Paeonia lactiflora* Extract on the mRNA Levels of TPH2, SERT, and ER*β* in the DRN of Rats

**Figure 4:**
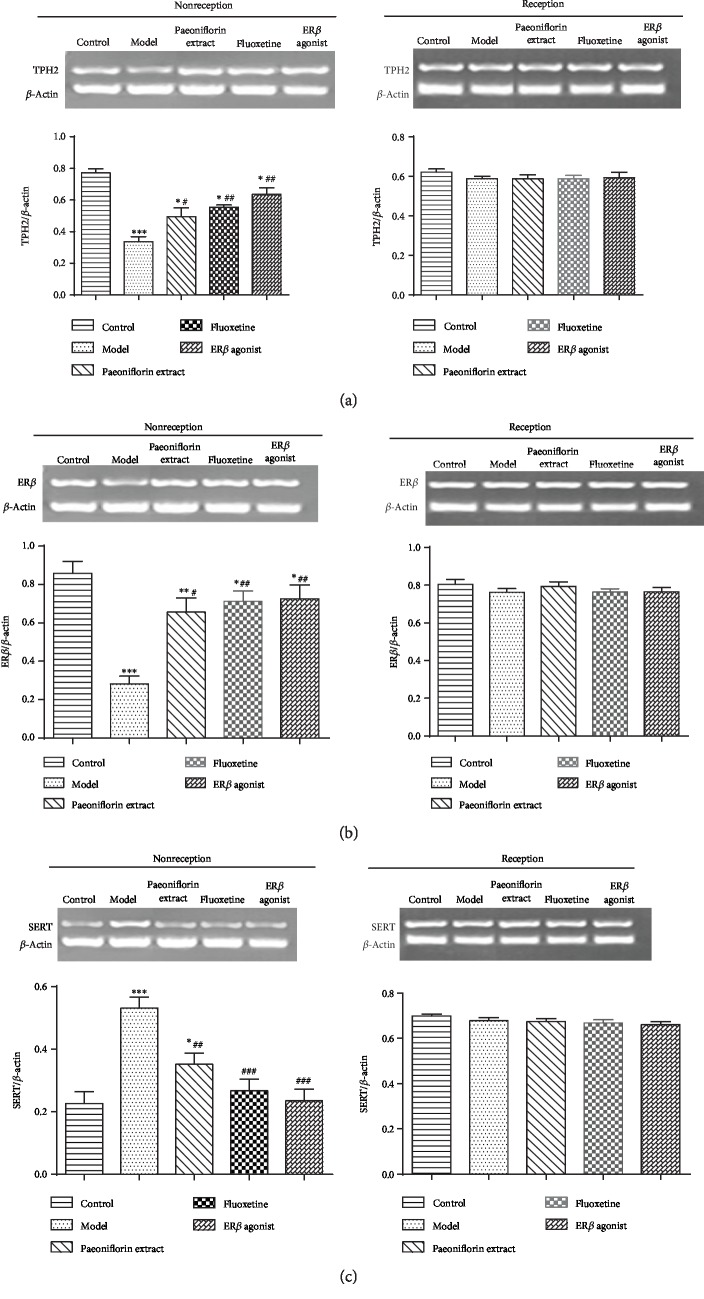
mRNA expression of *TPH2*, *SERT*, and *ERβ* in dorsal raphe nucleus in reception and nonreception stage. (a) *TPH2* mRNA level, (b) *ERβ* mRNA level, and (c) SERT mRNA level. The mRNA expression of related genes in control, model, *Paeonia lactiflora* extract, fluoxetine, and ER*β* agonist group were detected by RT-PCR. ^∗∗∗^*P* < 0.001 as compared with the control group; ^#^ and ^##^*P* < 0.05 and < 0.01 as compared with the model group, respectively.

The mRNA expression of *TPH2*, *SERT*, and *ERβ* in DRN of control, model, *Paeonia lactiflora* extract, fluoxetine, and ER*β* agonist groups were detected by RT-PCR. In the nonreception phase, the expression of *TPH2* and *ERβ* mRNA in each group was significantly decreased as compared with the control group (*P* < 0.01, Figures 4(a) and 4(b)). However, the expression of SERT mRNA was significantly increased (*P* < 0.01, Figure 4(c)). After intervention, the expression of *TPH2* and *ERβ* mRNA was significantly increased compared with the model group (*P* < 0.05, Figures 4(a) and 4(b)). The expression of *SERT* mRNA was significantly decreased (*P* < 0.05, Figure 4(c)). In the reception phase, there was no significant difference in the expression of *TPH2*, *SERT*, and *ERβ* mRNA in the DRN among each group (*P* > 0.05, Figures 4(a)–4(c)).

### 3.3. Effect of *Paeonia lactiflora* Extract on the Protein Expression of TPH2, SERT, and ER*β* in Rat DRN

**Figure 5:**
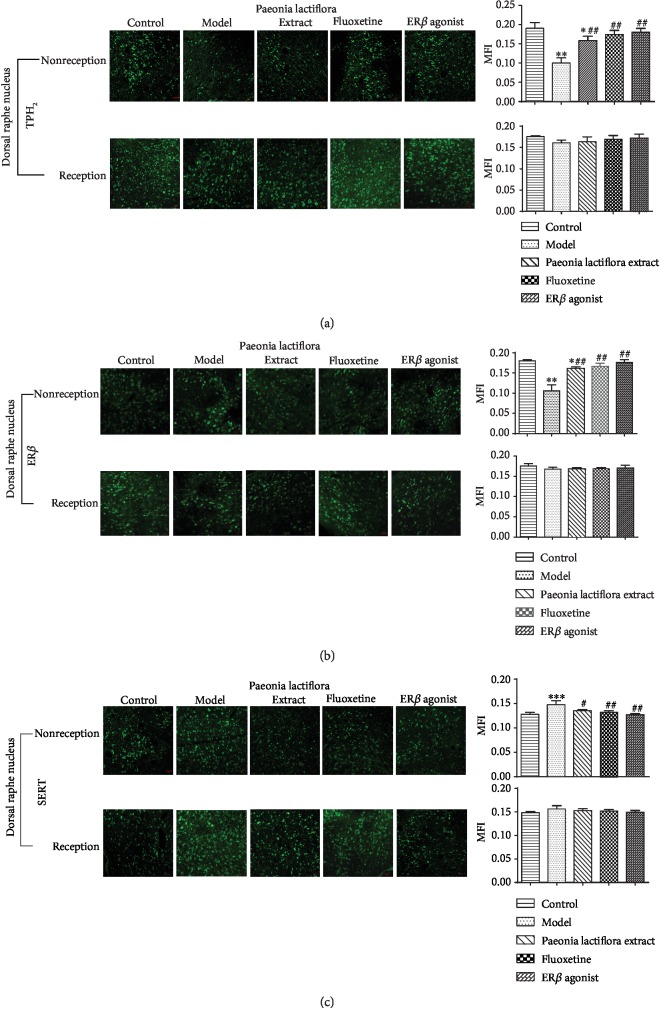
Neuronal distribution pattern and protein expression of TPH2, SERT, and ER*β* in dorsal raphe nucleus in reception and nonreception stage (IF 10x20). (a) TPH2 protein expression, (b) ER*β* protein expression, and (c) SERT protein expression. The protein expression of related genes in control, model, *Paeonia lactiflora* extract, fluoxetine, and ER*β* agonist group were detected by immunofluorescence. Left, neuronal distribution pattern; right, the fluorescence intensity. ^∗∗∗^*P* < 0.001 as compared with the control group; ^#^ and ^##^*P* < 0.05 and< 0.01 as compared with the model group, respectively.

The TPH2, SERT, and ER*β* distributions in the DRN of rats were detected by immunofluorescence. As shown in Figure 5, the TPH2, SERT, and ER*β*-positive cells in the DRN of each group were shown as green fluorescence. Morphology of TPH2 and SERT-positive cells was round or spotted. TPH2 and SERT proteins were mostly distributed in the cell membrane (Figures 5(a) and 5(b)). The morphology of ER*β*-positive cells was round or spotted. ER*β* protein was mostly distributed in the nucleus and sporadically distributed in the cell membrane (Figures 5(a) and 5(c)).

In the reception phase, the TPH2, SERT, and ER*β*-positive cells in the DRN had abnormal distribution and were more uniform and mostly oval after modeling. The cell distribution and morphology appeared normal after drug intervention. Compared with the normal group, the mean fluorescence intensity (MFI) of TPH2, SERT, and ER*β* in the DRN of the model group had no significant difference (*P* > 0.05). After drug intervention, the MFI of TPH2, SERT, and ER*β* in each group were not significantly different from those in the model group (*P* > 0.05). In the nonreception phase, the distribution of TPH2-positive cells became sparse, the number of cells decreased, and the morphology was mostly oval after modeling (Figure 5(a)). The number of SERT-positive cells increased, and the morphology was mostly oval (Figure 5(b)). The number of ER*β*-positive cells decreased and most cells were distributed on the cell membrane. Some cells were even dried up (Figure 5(c)). Compared with the control group, the MFI of TPH2 and ER*β* in the DRN of the model group was significantly reduced (*P* < 0.01, Figures 5(a) and 5(c)). The MFI of the SERT was significantly increased (*P* < 0.05). After intervention with drugs, the MFI of TPH2 and ER*β* in rats of each treatment group was significantly increased as compared with the model group. The MFI of SERT was significantly decreased (*P* < 0.05). There was no significant difference between the treatment groups (*P* > 0.05).

## 4. Discussion

In this study, the PMS anxiety model was established based on improved emotional stimuli and mainly through electrical stimulation. Rats of nonreception phase were placed in an electrically-stimulated cage with adjustable irritant, noise, and pulse. The electrical stimulation of the rat's foot was accompanied by noise interference. Noise stimuli were used to establish conditional reflexes in rats. Even if the current became smaller, the noise interference could still achieve the same stress effect as the current stimulation, which can strengthen the psychological stress while reducing the physical injury caused by the electric shock [5, 19]. It is reported that stress can easily lead to imbalance in the body and produce anxiety, depression, bipolar disorder, and other mental and emotional diseases [20].

In this experiment, the female rats of the Wistar germline with advanced functions of the pituitary-adrenal system and sensitive to stress were selected [21]. Female rats were ferocious and irritated after electrical stimulation, which facilitated the successful replication of the PSM model. The rats were housed in an adaptive environment for one week and kept in a state of daytime excitement using day-and-night reversed feeding. The rats used were preselected. The biological characteristics of the rats were generally consistent. The rats were scored according to the open field behavior test and vaginal smear. The rats with the similar scores in the open field and in the nonreception stage were selected to eliminate the effects of individual factors. Rat estrus cycle is divided into preestrus, estrus, late estrus, and estrus [22]. In reality, the proportion of cells in different periods is not easy to grasp, resulting in large subjective errors. Therefore, in this study, the rat estrous cycle is divided into two stages of reception stage and nonreception stage according to the detection of white blood cells [23]. The nonreception stage of rats is equivalent to the premenstrual stage, so as to ensure the reliability of the model preparation results.

The open field experiment is a classic behavioral experiment that can well evaluate the animal's emotional level and its exploration behavior in an open and unfamiliar environment [24]. It can be used to test the “excited” or “depressed” state of the animal's central nervous system. This study shows that the total distance of activity in the model rats significantly increased as compared with the control group, suggesting that the activity of the model rats increases significantly with obvious anxiety-like behavior. The open field experiment showed that the rat model of PMS anxiety was successfully prepared. After intervention with the *Paeonia lactiflora* extract, the total distance travelled by the rats was significantly lower than that of the model group, indicating that the *Paeonia lactiflora* extract is effective for PMS.

This study indicates that rats were less sensitive to external stimuli during reception stage, and no obvious abnormalities were observed in all indexes. The mRNA levels of ER*β* and TPH2 were downregulated and that of SERT was upregulated in rats during nonreception stage. Immunofluorescence showed that the ER*β* in the DRN of PMS anxiety rats was significantly decreased and TPH2 and SERT showed corresponding abnormal expression. These results indicate that the anxiety of PMS rats may be due to the downregulation of ER*β* expression, which leads to the downregulation of TPH2 and increased expression of SERT, thus resulting in reduced 5-HT level and causing anxiety. This may be one of the important links in the pathogenesis of PMS anxiety. After intervention with the *Paeonia lactiflora* extract, the number, morphology, and protein expression of positive cells of ER*β*, TPH_2_, and SERT were restored and close to the control group, demonstrating that *Paeonia lactiflora* extract can regulate PMS anxiety.

## 5. Conclusions

In conclusion, the model of PMS anxiety is successfully prepared by using multifactorial modeling based on emotional stimuli. *Paeonia lactiflora* extract can upregulate the expression of ER*β* and TPH2 in the DRN and downregulate the expression of SERT, thus regulating PMS anxiety. The mechanism underlying the effect of *Paeonia lactiflora* extract in PMS anxiety needs further study.

## Figures and Tables

**Table 1:** Sequences of primers used for RT-PCR.

Gene	Sequence (5′-3′)
*TPH2*	Forward: TTGGGAGGTGGTTTCTACTTTC
	Reverse: TGTTTCTCTGTGACTCGGTTTC3

*SERT*	Forward: CCCTCTGTTTCTCCTGTTCATC
	Reverse: CTGAGAGTCCACGGAAAGAAGT

*ERβ*	Forward: CGTCAGGCACATCAGTAACAAG
	Reverse: GGACAATCCTTCCAAATCAGAC

*β-Actin*	Forward: CACCCGCGAGTACAACCTTC
	Reverse: CCCATACCCACCATCACACC

## Data Availability

The data used to support the findings of this study are available from the corresponding author upon request.
